# P-2002. Incidence and Risk of COVID-19 Hospitalization Among Unvaccinated Children

**DOI:** 10.1093/ofid/ofae631.2159

**Published:** 2025-01-29

**Authors:** Ousseny Zerbo, Julius Timbol, John R Hansen, Kristin Goddard, Evan Layefsky, Pat Ross, Bruce Fireman, Dao Nguyen, Tara L Greenhow, Nicola P Klein

**Affiliations:** Division of Research Kaiser Permanente Vaccine Study Center, Oakland, CA; Kaiser Permanente Vaccine Study Center, Oakland, CA; Division of Research Kaiser Permanente Vaccine Study Center, Oakland, CA; Division of Research Kaiser Permanente Vaccine Study Center, Oakland, CA; Division of Research Kaiser Permanente Vaccine Study Center, Oakland, CA; Kaiser Permanente Vaccine Study Center, Oakland, CA; Division of Research Kaiser Permanente Vaccine Study Center, Oakland, CA; Kaiser Permanente Northern California, San Jose, California; Kaiser Permanente Northern California, San Jose, California; Division of Research Kaiser Permanente Vaccine Study Center, Oakland, CA

## Abstract

**Background:**

Although most children often experience mild illness following SARS-CoV-2 infection, some can develop severe disease requiring hospitalization, admission into an intensive care unit (ICU), or die.

**Methods:**

Children aged 0 - < 18 years, members of Kaiser Permanente Northern California (KPNC), an integrated healthcare organization with more than 4 more million members, were followed from March 1, 2020, until the earliest occurrence of: chart-confirmed COVID-19 hospitalization, disenrollment from KPNC, age 18 years, receipt of COVID-19 vaccine, death, or study end (December 31, 2022). We calculated the incidence rate of hospitalization by SARS-CoV-2 variant period and by age group. We determined risk factors for hospitalization using Poisson regression. We also conducted descriptive analyses of hospitalized cases.

**Results:**

Among 1,107,799 children, 423 were hospitalized for COVID-19 during follow-up. The incidence of hospitalization increased with each new SARS-CoV-2 variant and was highest among children aged < 6 months. Among the < 6-month-olds, the incidence rate per 100,000 person-months was 7 during pre-delta, 13.3 during delta and 22.4 during omicron. Black (RR= 2.05, 95% CI: 1.33 – 3.16) and Hispanic children (RR = 1.82, 95% CI: 1.34 – 2.46) and children with any comorbidities were at high risk of hospitalization (RR = 3.81, 95% CI: 2.94 – 4.95). Overall, 20.3% of hospitalized children were admitted to an intensive care unit (ICU), but ICU admission was 36.1% among 12 - < 18-year-olds. The majority of ICU admits (91.8%) had no comorbidities.

**Conclusion:**

Children too young to be vaccinated had the highest incidence of COVID-19 hospitalization, while adolescents had the highest proportion of ICU admissions. To prevent severe disease in children and adolescents, everyone eligible should be vaccinated.

**Disclosures:**

Nicola P. Klein, MD, PhD, CSL Seqirus: Grant/Research Support|GlaxoSmithKline: Grant/Research Support|Merck: Grant/Research Support|Pfizer: Grant/Research Support|Sanofi Pasteur: Grant/Research Support

